# Atrial Fibrillation and Diabetes Mellitus: Dangerous Liaisons or Innocent Bystanders?

**DOI:** 10.3390/jcm12082868

**Published:** 2023-04-14

**Authors:** Ana Lorenzo-Almorós, Jesús Casado Cerrada, Luis-Antonio Álvarez-Sala Walther, Manuel Méndez Bailón, Óscar Lorenzo González

**Affiliations:** 1 Internal Medicine Department, Hospital General Universitario Gregorio Marañón, 28007 Madrid, Spain; 2 Department of Medicine, School of Medicine, Universidad Complutense de Madrid, 28007 Madrid, Spain; 3 Internal Medicine Department, Hospital Universitario de Getafe, 28095 Madrid, Spain; 4 Internal Medicine Department, Hospital Universitario Clinico San Carlos, 28040 Madrid, Spain; 5 Laboratory of Diabetes and Vascular Pathology, Instituto de Investigaciones Sanitarias-Fundación Jiménez Díaz, Universidad Autónoma, 28040 Madrid, Spain; 6 Biomedical Research Centre in Diabetes and Associated Metabolic Disorders (CIBERDEM) Network, 28040 Madrid, Spain

**Keywords:** atrial fibrillation, diabetes mellitus, atrial remodeling, thrombotic risk

## Abstract

Atrial fibrillation (AF) is the most common arrhythmia in adults and diabetes mellitus (DM) is a major risk factor for cardiovascular diseases. However, the relationship between both pathologies has not been fully documented and new evidence supports the existence of direct and independent links. In the myocardium, a combination of structural, electrical, and autonomic remodeling may lead to AF. Importantly, patients with AF and DM showed more dramatic alterations than those with AF or DM alone, particularly in mitochondrial respiration and atrial remodeling, which alters conductivity, thrombogenesis, and contractile function. In AF and DM, elevations of cytosolic Ca^2^⁺ and accumulation of extra cellular matrix (ECM) proteins at the interstitium can promote delayed afterdepolarizations. The DM-associated low-grade inflammation and deposition/infiltration of epicardial adipose tissue (EAT) enforce abnormalities in Ca^2+^ handling and in excitation-contraction coupling, leading to atrial myopathy. This atrial enlargement and the reduction in passive emptying volume and fraction can be key for AF maintenance and re-entry. Moreover, the stored EAT can prolong action of potential durations and progression from paroxysmal to persistent AF. In this way, DM may increase the risk of thrombogenesis as a consequence of increased glycation and oxidation of fibrinogen and plasminogen, impairing plasmin conversion and resistance to fibrinolysis. Additionally, the DM-associated autonomic remodeling may also initiate AF and its re-entry. Finally, further evidence of DM influence on AF development and maintenance are based on the anti-arrhythmogenic effects of certain anti-diabetic drugs like SGLT2 inhibitors. Therefore, AF and DM may share molecular alterations related to Ca^2+^ mobility, mitochondrial function and ECM composition that induce atrial remodeling and defects in autonomic stimulation and conductivity. Likely, some specific therapies could work against the associated cardiac damage to AF and/or DM.

## 1. Atrial Fibrillation and Diabetes Mellitus

### 1.1. Epidemiologic Interaction of Atrial Fibrillation and Diabetes

Atrial fibrillation (AF) is the most common heart arrhythmia that can present as paroxysmal or persistent [1]. Approximately forty-three million people can suffer from AF and its estimated prevalence in adults can reach 1–4% of the population [2]. These data could be doubled in the next decades due to the increasing life-span and to the discovery of new diagnostic approaches [2]. AF entails a true threat for patients due to its dramatic associated morbidity and mortality [3,4]. It can be linked to a five-fold increased risk of stroke and three-fold elevated risk of heart failure [5]. Indeed, AF can be considered a cardiac biomarker of advanced atrial myopathy closely related to ventricular myopathy and specifically, heart failure with preserved ejection fraction (HFpEF) [6]. Consequently, in elderly patients (>75 years-old) the annual rate of mortality derived from AF reaches 15% [7]. Importantly, modifiable risk factors of AF include hyperthyroidism, chronic kidney disease, high blood pressure, cardiomyopathies (heart failure, ischemic heart disease), obesity, and diabetes mellitus (DM). In this regard, the prevalence of DM in patients with AF reaches 23–40%, and also, 15% of DM patients suffer from AF [8,9,10].

DM is a major cardiovascular risk factor associated with the occurrence of cardiovascular events such as ischemic heart disease, heart failure, stroke, peripheral artery disease, or chronic kidney disease [4]. In 2017, 462 million individuals (6.3% of the population) were affected by type 2 diabetes (T2DM) and this prevalence is predicted to spread up to 7% in 2030 [11]. In the Framingham Heart Study, DM was associated with a 40% higher probability of AF in men, and with 60% in women [12]. In some meta-analyses, a 20% elevated risk of AF was observed in pre-diabetic subjects while a 28–40% increase was found after DM [13,14]. Although these studies mainly referred to T2DM, other reports showed a similar tendency for type-1 diabetic patients [15]. In fact, poor glycemic control and glycemic fluctuations can also elevate the risk of AF [16,17,18]. In the ARIC (Atherosclerosis Risk in Communities) study, each 1% increase in glycated hemoglobin (HbA1c) levels was related to a 13% higher rate of AF [19]. Similarly, Aune et al. [14] described a 12% elevation in the relative risk of AF per 20 mg/dl increase in blood glucose. In addition, AF and DM share several risk factors and comorbidities. Hypertension, obesity, dyslipidemia, gut dysbiosis, inflammatory diseases, fatty liver disease, coronary artery disease, chronic kidney disease, and obstructive sleep apnea are frequently present in both pathologies [1,4]. Thus, DM could strengthen AF symptoms and its derived hospitalization and mortality rates [10].

### 1.2. Interactive Mechanisms of Cardiac Diabetes and Atrial Fibrillation

**Figure 1:**
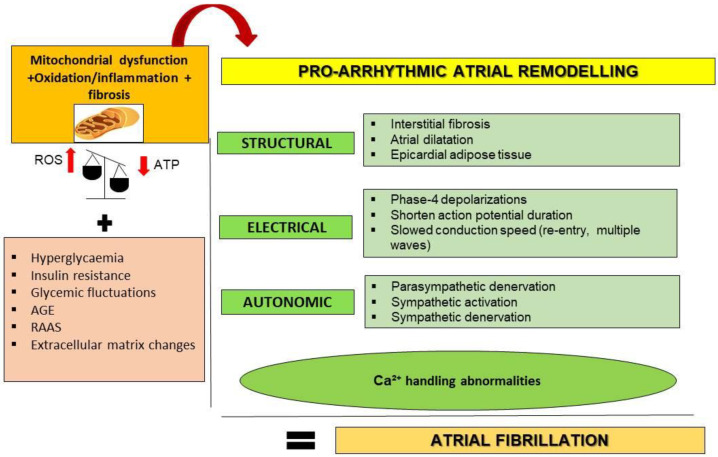
**Proposed mechanisms linking T2DM and AF through atrial remodeling**. T2DM may favor pro-arrhythmic atrial remodeling through oxidative stress, inflammation, and mitochondrial dysfunction as key mechanisms. Additionally, hyperglycemia, insulin resistance, glucose fluctuations, AGE (advanced glycation end products), renin–angiotensin–aldosterone system (RAAS) overactivation, and deposition of extracellular matrix may contribute. Pro-arrhythmic atrial remodeling components include structural, electrical, and autonomic alterations. Calcium (Ca^2^⁺) handling abnormalities could interconnect and originate all components. ROS, reactive oxygen species; ATP, adenosine triphosphate; SR, sarcoplasmic reticulum; IcaL, L-type Ca^2^⁺ current.

AF originates from the presence of ectopic foci of depolarization in atrial cardiomyocytes. This supra-ventricular tachyarrhythmia may transform atrial contractions into inefficient movements [1]. Additionally, the altered electrophysiological properties and areas of structural remodeling trigger spontaneous activators and re-entrant drivers [1]. Interestingly, some underlying mechanisms including mitochondrial dysfunction, oxidation, fibrosis, inflammation, fatty accumulation and filtration, calcium impairment, and thrombogenesis can be shared and enforced by DM after specific structural, electrical, and autonomic remodeling [20,21,22] (Figure 1).

(i) Mitochondrial dysfunction and oxidative stress

In healthy cardiomyocytes, ATP is mainly obtained through oxidative phosphorylation (OXPHOS) in the mitochondria, and only a low amount is derived from the glycolytic pathway, outside the mitochondria [23]. Fatty acids are the main metabolic substrates which produce 60–90% of total ATP, whereas glucose is used to a lesser extent (10–40%) by the heart [24]. However, under cardiac insults such as AF, cardiomyocytes may increase the energy demands of ATP and change the energy source. The rapid and uncoordinated electrical activity observed in the atria of patients with AF can shift OXPHOS to glycolysis by decreasing the level of respiratory complexes [25]. This chronic response can lead to mitochondrial failure and inefficiency of ATP consecution, which triggers atrial remodeling and dysfunction [26,27,28]. In consequence, excessive generation of ROS during AF is associated with ER stress, which activates the unfolded protein response (UPR) and the proinflammatory nuclear factor-kappa B (NF-κB) and NLRP3 signaling [29] (see later). As part of the self-healing capacity of the human body, some small defects can be remodeled or regenerated by resident cells. However, not all defects can properly regenerate, which is especially true for the human heart. This remodeling affects all cardiac (cardiomyocytes, fibroblasts) and pericardial (adipocytes, pericytes, smooth muscle, and endothelial) cells and extracellular matrix (ECM), producing atrial dilation and fibrosis, which delay electromechanical conduction [1]. Indeed, in paroxysmal AF, the stimulation of atrial remodeling is essential to progress to persistent AF [30].

**Figure 2:**
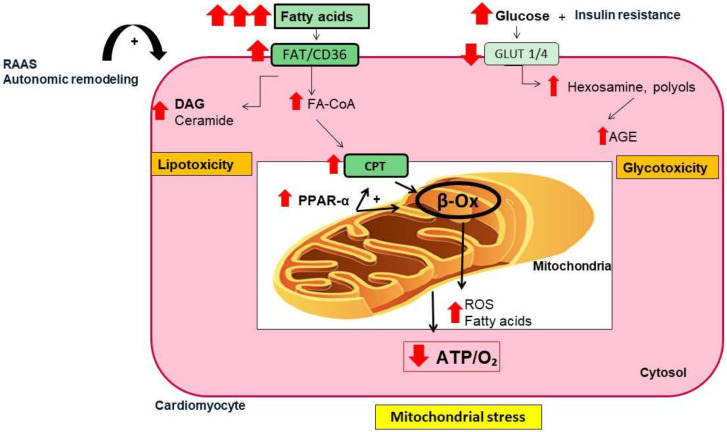
**Metabolic alterations in cardiac cells in T2DM.** In T2DM, insulin resistance reduces the expression of glucose transporters (i.e., Glut-1, Glut-4) in cardiac cells, leading to reduction in glucose assimilation, hyperglycemia, and excessive utilization of fatty acid as a main energetic substrate. These responses activate the nuclear peroxisome proliferator-activated receptor-alpha (PPAR-α), which upregulates specific proteins involved in fatty acid transport (i.e., CPT) and β-oxidation (β-ox). The overload of fatty acid saturates β-ox, leading to accumulation of ceramide and reactive oxygen species (ROS) that increase oxidative stress and lipotoxicity. Additionally, non-degraded glucose generates glycolytic metabolites (i.e., advanced glycated end products (AGEs), polyols, and hexosamine) contributing to glucotoxicity. Both glucotoxicity and lipotoxicity favors mitochondrial stress that affects mitochondrial dynamics and regeneration, as well as energetic consecution (ATP/O_2_ ratio). Finally, the overactivation of the renin–angiotensin–aldosterone system (RAAS) and the autonomic remodeling may enhance insulin resistance, inflammation, ROS generation, and mitochondrial stress. FAT/CD36, fatty acid translocase; DAG, diacylglycerol; Fa-CoA, fatty acyl-coenzyme A; CPT, carnitine palmitoyl-transferase; ATP/O_2_, adenosine triphosphate/oxygen ratio.

T2DM is characterized by insulin resistance and subsequent hyperglycemia derived from downregulation of glucose transporters (i.e., Glut4 and Glut1 in cardiac cells). The use of fatty acids as a main source of ATP leads to inefficient production of energy and metabolic inflexibility during DM [31]. Fatty acid activates the nuclear peroxisome proliferator-activated receptor-alpha (PPAR-α) to upregulate fatty acid carriers and β-oxidation enzymes [32]. The excessive fatty acid uptake can also saturate β-oxidation and contribute to fatty acid transformation toward lipotoxic metabolites, such as ceramide and diacylglycerol. It also provokes the massive release of reactive oxygen and nitrogen species (ROS and NOS) that favors oxidative stress. Moreover, the non-utilized glucose generates advanced glycated end products (AGE) and other glucose-metabolites such as hexosamine and polyols that promote glucotoxicity and an oxidative milieu [33,34]. Mitochondrial-derived ROS also activates NADPH oxidase, driving a vicious cycle of ROS production, which accelerates myocardial injury and atrial remodeling [35,36] (Figure 2*)*. It can also impair mitochondrial biogenesis and autophagy, and disturb fission and fusion balance, leading to deposition of fragmented dysfunctional mitochondria and chronic low-grade inflammation [27,37]. Thus, T2DM favors mitochondrial stress, and contractile dysfunction in the atria. In fact, patients with T2DM and AF showed more dramatic alterations in mitochondrial OXPHOS, complex assembly, and oxidation than those with AF only [38]. In an experimental model of DM, antiarrhythmic drugs that improved mitochondrial function, also alleviated atrial remodeling and AF incidence [39].

(ii) Inflammation and fibrosis

Histologic analysis of AF hearts exhibited immune cell (e.g., leukocytes and especially, macrophages) infiltration and low-grade inflammation [40]. Additionally, the inflammasome protein complex, which is an intracellular multimeric structure in immune and non-immune cells that initiates inflammatory signaling, was implicated in AF pathogenesis [41]. Activation of the NLRP3 inflammasome in atrial cardiomyocytes after hypertension, diabetes, and obesity was also detected as a pathogenic response in paroxysmal, chronic, and postoperative AF [42]. NLRP3 also turned on other inflammatory cytokines such as IL1β, TNF-α, and IL-6 [43], which promoted AF through atrial dilation [44,45]. Interestingly, NLRP3 activation was associated with aberrant release of CM-Ca2þ from the SR, and with atrial hypertrophy and shortening of the effective refractory period [46]. In diabetic patients with AF, the NLRP3–CASP1–galectin 3 signaling was stimulated in atrial tissue, contributing to AF onset [47].

In the myocardial tissue, there is an interplay between inflammation and fibrosis. Both responses are major pathophysiological mechanisms operating after AF and DM. Cardiac fibrosis is a process of pathological remodeling that induces abnormalities in matrix composition and quality, as well as in muscle function. Due to mitochondrial failure, Ca^2^⁺ transport between cytosol and mitochondria can be impaired (see later). Elevations of cytosolic Ca^2^⁺ in cardiac fibroblasts promotes proliferation and differentiation into myofibroblasts, which can release extra cellular matrix (ECM) proteins to the interstitium [48]. The excessive and continuous deposition of ECM proteins (i.e., collagen type-I) generates fibrotic scars, which cause reduction in the ejection fraction due to a stiffened myocardial matrix and impaired electric conductance [49]. In particular, atrial fibrosis is also highly dependent on the epicardial expansion of the atria following de novo generation of myofibroblast [50]. This atrial enlargement is a key factor for AF maintenance and re-entry [51]. Interestingly, epicardial precursors from atrial appendages can differentiate into myofibroblasts when obtained from patients with AF [52]. In addition, under a DM environment, interstitial fibrosis is one of the first remodeling changes that promotes AF [53]. The disrupted fiber continuity and the cell-to-cell coupling hinder action potential propagation and promote re-entrant excitation for AF [22,54,55]. Additionally, local angiotensin-II activates the NF-κB pathway and myofibroblast differentiation, which overexpress transforming growth factor-β (TGF-β) and ECM proteins [56]. Key mediators of arrhythmogenic remodeling in DM patients include oxidative stress and inflammatory molecules [57]. These alterations in atrial ultrastructure have also been linked to AF inducibility in DM rats [35].

(iii) Calcium disruption

In cardiomyocytes, Ca^2^⁺ is released from the SR to cytosol after membrane depolarization. Ca^2^⁺ activates calmodulin kinase type II (CaMKII) and hyper-phosphorylates ryanodine receptors. However, during AF, cytosolic accumulation of Ca^2+^ over-activates CaMKII and increases Na⁺/Ca^2^⁺ exchangers that cause delayed atrial afterdepolarizations (named phase-4 depolarizations), propagating wavefronts that initiate a spontaneous premature ectopic activity [22]. The slowed conduction also causes wavelength shortening, which encourages stability of AF circuits [22]. Then, persistent AF is maintained by complex re-entrant excitation circuits and multiple circulating wavelets [22,58]. In addition, CaMKII activates the calcineurin/nuclear factor of activated T cells (NFAT) signaling, which reduces the L-type Ca^2^⁺ current and duration of the action potential, facilitating AF re-entry [53,58,59,60]. Thus, AF can be by itself, a stimulus of atrial remodeling and AF perpetuation (“AF begets AF”) [61]. More interestingly, DM may also induce chronic phase-4 depolarizations. Both the small conductance Ca^2^⁺-activated K⁺ (SK) and the inward rectifier current Ik1 (IkAChc) channels, which maintain AF and re-entry to AF, are upregulated under DM [58,62,63,64]. Specific proteins of the Gap junction channels, such as connexin-40 and -43, involved in AF remodeling, can also be altered [65,66]. Moreover, DM subjects show reductions in passive emptying volume and emptying fraction that elicit arrhythmogenic changes [67].

(iv) Fatty acid accumulation and infiltration

The abundance of epicardial adipose tissue (EAT) around the atria is associated with an increased risk of AF. Fat deposits around the heart initially serve as a fatty acid (FA) store for energy supply. The EAT is located between the surface of the myocardium and the pericardium, surrounding and in direct contact with the major coronary arteries. However, excessive expansion of the EAT depot has been associated with chronic inflammation and heart disease [68]. Overload of fat alters Ca^2+^ handling and excitation–contraction coupling [69]. In T2DM, usually associated with obesity, these visceral adipose stores are increased. Lipid accumulation causes cell enlargement, which decreases oxygen delivery [70]. The hypoxic and inflammatory environment is specifically implicated in the pathogenesis of diastolic dysfunction [68]. On atria, deposited EAT encourages remodeling actions that promote atrial myopathy [71,72]. On ventricles, accumulated EAT favors reduction in distensibility and increased left volume filling pressures, which triggers HFpEF [73].

In addition, EAT becomes an endocrine tissue impacting on AF development. The thickened EAT is dysfunctional and releases adipokines, pro-oxidant, inflammatory, and fibrotic factors that induce myocardial alterations and coronary artery disease [74,75,76,77]. These alterations provoke energetic inefficiency and ROS, which stimulate the release of proapoptotic proteins such as cytochrome c and caspases-3 and -9 [78]. In addition, the accumulated EAT may initiate early and delayed afterdepolarizations and serve as a mechanical obstacle to cardiac excitation [74]. Its adipocytes can infiltrate into the myocardium to provoke non-uniform anisotropic propagation of the activated wave front, transforming electrical impulses to *“zigzag”* paths that pushes re-entry to AF [79]. Moreover, during AF, epicardium is activated and epicardial-derived cells, preprogrammed towards a specific cell fate, can contribute to fibro-fatty infiltration into the sub-epicardium area. Herein, this adipose tissue can be replaced by fibrosis, favoring electrical dissociation between sub-epicardium and sub-endocardium areas, and focal fibrillation waves [50]. Thus, fibro-fatty infiltration can also provide the substrate of AF. Finally, the stored EAT is able to prolong action potential durations and progression from paroxysmal to persistent AF [80]. In fact, EAT thickness has been associated with P-wave dispersion as a predictor of arrhythmogenesis and adverse outcomes [81,82,83].

(v) Autonomic remodeling

The autonomic remodeling appears in three consecutive stages, initially as parasympathetic denervation, followed by sympathetic hyperactivation, and finally, as sympathetic denervation [84]. Parasympathetic stimuli tend to favor macro-re-entry phenomena, whereas sympathetic stimuli promotes abnormal automaticity and triggered neuronal activity [85]. The hyperactivity of the cardiac autonomic nervous system (ANS) is critical for the origination and maintenance of AF. ANS can initiate AF, and AF further enhances the activity of the cardiac ANS. Autonomic hyperinnervation may trigger an autonomic reflex to reduce the vagal and sympathetic nerve activity as a protective mechanism. However, hyperinnervation and sprouting of the atrial autonomic nerves, working synergistically with electrical remodeling, are key for perpetuating AF [86]. Interestingly, autonomic remodeling is often present in DM subjects even before DM diagnosis [87]. Evidence of autonomic dysfunction, quantified by heart rate variability or heart rate recovery, have been reported in diabetic individuals [88,89]. The release of acetylcholine was decreased in atrial appendage samples from elderly DM patients, suggesting impaired vagal responses attributed to a defect in ganglionic transmission or to a loss of preganglionic vagal efferent nerve fibers [90]. Studies in T2DM murine models also described an impaired parasympathetic tone of preganglionic vagal neurons and nerve fibers, and negative chronotropic responses [91]. Thus, DM-associated autonomic remodeling may also regulate AF initiation and maintenance through modulation of cardiac electricity [92].

(vi) Thrombogenesis

**Figure 3:**
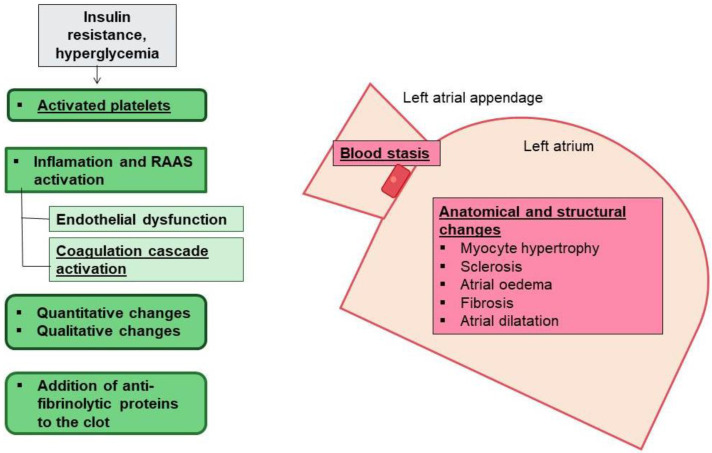
**AF, T2DM, and thrombotic risk**. In AF there are anatomical characteristics such as the presence of left atrial appendage, and structural changes in the left atrium that predispose to blood stasis and thrombogenesis (pink boxes). T2DM also triggers inflammation, endothelial dysfunction and activated platelets, and alters coagulation factors and fibrinolysis that contribute to the increased risk of thrombogenesis.

Intriguingly, atrial remodeling was described by Masawa et al. as a granular and wrinkled endocardium appearance associated with edematous and fibrous thickening, with small areas of endothelial denudation and thrombotic aggregations [93]. The thrombotic risk has been traditionally attributed to blood stasis in the left atrium after disruption of atrial systole (Figure 3). After AF, the anatomical and structural modifications may predispose the heart to thrombogenesis [94]. However, evidence supports the existence of an underlying pro-thrombotic state in patients with AF [95]. Accumulation of ECM proteins affects blood stasis. In patients with AF, abnormal coagulation components such as increased fibrin turnover and the thrombin/antithrombin index has been reported. These factors overexpress tissue-plasminogen activator (t-PA) antigen and enhance the plasmin/antiplasmin ratio [96]. Moreover, the presence of left atrial dilation, abnormalities in diastolic filling, and the existence of a blind ended passage (appendage) in the left atrium, contribute to increased risk of thrombus formation and thromboembolic events during AF [97,98]. Furthermore, AF patients also exhibit platelet activation [99]. The activated platelets exhibit a hyperreactive phenotype with upregulation of CD31, CD26P and CD63, and platelet surface receptors (i.e., GPIb and GPIIb/IIIa), which enhance cell adhesion and aggregation [100,101].

Importantly, in this scenario, DM may increase the risk of thrombogenesis as a consequence of inflammation, atrial myopathy, and EAT expansion [26] (Figure 3). Hyperglycemia and insulin resistance are both associated with increased early platelet activation [102,103]. The DM-associated low-grade inflammation stimulates thrombogenesis through IL-6 production to enhance thrombin sensitivity, fibrinogen and tissue factor expression, and endothelial damage [104,105,106,107,108]. The endothelial dysfunction encourages expression of adhesion molecules to attract inflammatory and foam cells as a part of the atherosclerosis phenomenon. When atheroma plaques rupture, a pro-thrombotic core is exposed, activating more platelets and release of pro-coagulation factors [109]. Insulin resistance also decreases nitric oxide production, enhancing vasoconstriction [110,111]. Moreover, higher levels of PAI-1 and lower expression of anticoagulant molecules, such as thrombomodulin and protein C, have been described in DM patients [112,113]. In addition, the glycation and oxidation of fibrinogen and plasminogen impairs plasmin conversion and supports denser fibrin networks, increasing resistance to fibrinolysis [114,115,116]. In consequence, DM correlates with a 70% relative increase in risk of stroke caused by the presence of atrial myopathy after atrial remodeling, blood stasis, and thrombus formation [117,118,119].

### 1.3. Anti-Diabetic, Anti-Thrombotic, and Atrial Fibrillation (Table 1)

More evidence of the potential influence of DM on the development and maintenance of AF is based on the anti-arrhythmogenic effects of certain anti-diabetic drugs. Since most anti-diabetics affect atrial remodeling, they could be useful to attenuate AF progression. Metformin has been consistently demonstrated as an AF protector in different studies. In a Taiwanese cohort of early stage T2DM patients, metformin showed a reduction in the risk of AF for at least two years [120]. A similar result was observed in the amelioration of atrial flutter and supraventricular arrhythmia [121]. Metformin may exert its anti-AF effects through decreasing oxidative stress and atrial remodeling. In fact, in cultured atrial myocytes metformin inhibited ROS generation and myofibril degradation after induced-tachycardia [120]. Other anti-diabetics like the PPAR-α agonists thiazolidinediones could lower AF hazard by controlling atrial inflammation and fibrosis [57,122,123]. Dipeptidyl peptidase inhibitors (DPP-4i) were also able to decrease the risk of AF in the same cohort of Taiwanese patients with T2DM [124], but this effect was neutral in other trials [125,126,127]. Glucagon-like peptide-1 receptor agonists (GLP-1 RA) could diminish AF and atrial flutter in diabetic patients [128], but these actions could not be confirmed in meta-analysis [129]. Albiglutide was even related with a higher risk of AF in T2DM individuals [130]. Finally, the use of sulfonylureas and insulin could be controversial since they stimulate sympathetic signaling [57,121,126,131,132].

In contrast, new cardiovascular therapies such as the SGLT2 inhibitors (SGLT2i) may be more promising. A sub-study of the EMPA-REG OUTCOME (Empagliflozin Cardiovascular Outcome Event Trial in Type 2 Diabetes Mellitus Patients) demonstrated that patients with AF versus no AF benefited more with empagliflozin in relation to heart failure-related outcomes [133]. SGLT2i were also correlated with lower risk of AF, atrial flutter and sudden cardiac death in patients with T2DM [134]. In a head-to-head assay, administration of SGLT2i, but not DPP-4i, was linked with reduced re-entrant AF [135]. After meta-analysis, several SGLT2i decreased AF and atrial flutter (19.33%), with dapagliflozin being the most favorable [136]. Indeed, in a sub-analysis from the DECLARE-TIMI 58 (Dapagliflozin Effect on Cardiovascular Events-Thrombolysis in Myocardial Infarction 58) trial, dapagliflozin versus placebo reduced by 19% and 23% the risk of first AF/atrial flutter or the number of total AF/flutter events, respectively, in patients with T2DM and cardiovascular injury [137]. Nevertheless, in the Dapagliflozin in Patients with Heart Failure and Reduced Ejection Fraction (DAPA-HF) study, dapagliflozin did not ameliorate the risk of new-onset AF [138]. Importantly, Okunrintemi et al. reported a similar effect on risk of AF in patients with or without DM coexistence [139]. Thus, the anti-AF actions of SGLT2i could not be strictly dependent on glucose control. In this sense, SGLT2i might lessen electrical and structural atrial remodeling through improvements in mitochondrial function [140,141,142]. They could also help to moderate atrial dilatation through blood-pressure reduction, natriuresis, and diuresis [143], and ameliorate the risk of supraventricular ectopy and sinus node automatism by reabsorbing Mg^2+^ from the kidney [144,145]. Moreover, SGLT2i diminished uric acid levels, which have been linked with AF development [146,147], and decreased reticulated platelets as key cells for thrombus generation [148].

**Table 1:** Anti-diabetic treatments and atrial fibrillation. Some anti-diabetic drugs may influence the AF risk. Metformin and glitazones (i.e., pioglitazone), SGLT2 inhibitors and GLP-1 RA (* except for albiglutide) have demonstrated positive actions against AF, while sulfonylurea and insulin could induce deleterious effects. DPP-4 inhibitors may play neutral roles on AF risk.

Anti-Diabetic Drug	Beneficial Effect in AF	Neutral Effect in AF	Deletereous Effect in AF
**Metformin**	✓		
**Glitazones**	✓		
**Sulfonylurea**			✓
**DPP-IV inhibitors**		✓	
GLP-1 RA *	✓		
**SGLT2 inhibitors**	✓		
**Insulin**			✓

In addition, the risk of stroke varies considerably across different groups of patients with AF. Antiplatelets and anticoagulants are both antithrombotic drugs to prevent clot formation and thrombosis. Antiplatelets prevent platelets from clumping and forming a clot, while anticoagulants inhibit clotting factors of the coagulation cascade. The former is used for conditions that involve endothelial damage and platelets sticking to the injured site. In contrast, the latter are used for conditions that involve stasis, which causes blot clots (thrombosis). Since AF promotes stasis in the heart, anticoagulants are the preferential pharmacological therapy. However, there is not an established anti-thrombotic regimen for diabetic patients with AF. Besides achieving an optimal glycemic control, partially responsible for platelet activation and fibrinolysis [103], some anti-thrombotic drugs can be recommended. In clinical studies comparing non-vitamin-K oral anticoagulants (NOACs) with warfarin such as the ROCKET-AF (Rivaroxaban), ARISTOTLE (Apixaban), RE-LY (Dabigatran) and ENGAGE-AF (Edoxaban) a significant reduction in risk of stroke/systemic embolism was higher for NOACs but independent of DM presence [149]. However, diabetic subjects showed greater absolute benefits because of their intrinsic increased risk of stroke/embolism compared to non-diabetics [150].

## 2. Future Perspectives

Nowadays, the epidemiological links between AF and diabetes mellitus are only supported by smaller observations that provide insight into pathogenesis. Further and larger studies in humans will be welcomed. Additionally, the causal link between AF and DM could involve various pathologies such as hypertension, coronary artery disease or autonomic deregulation, but the possibility remains that diabetes may directly affect the atrial tissue, and AF could be a genuine trigger for hyperglycemia and insulin resistance. Moreover, DM may mask the cardiac symptoms of the first-recorded episode of AF, possibly because of diabetic neuropathy [151]. Thus, the impact of known complications of DM on electrophysiological properties of atrial myocardium must be studied. In this sense, DM-associated gut dysbiosis has been unveiled as a direct contributor to AF. Some gut microbiota-derived metabolites can promote and regulate AF [152]. Lipopolysaccharide, indoxyl sulfate and secondary bile acids triggered atrial remodeling by increasing afterdepolarizations and by reducing conduction velocity. In addition, trimethylamine N-oxide (TMAO) emphasized autonomic remodeling by stimulation of sympathetic activity [153]. Interestingly, activation of the NLRP3 inflammasome, cytokine release and mitochondrial dysfunction were common mechanisms of action for these stimuli. Along these lines, other concomitant stimuli such as alterations in circadian patterns could promote AF development in diabetic patients. Intense anti-diabetic treatment before lunch and patterns of insulin secretion and resistance may lead to glycemic fluctuations and autonomic dysfunction, leading to AF [154].

## 3. Conclusions

DM may influence AF development indirectly, as a contributor with other cardiovascular risk factors, but also directly and independently. A combination of structural, electrical, and autonomic remodeling may lead to AF, which may constitute the final step of atrial remodeling. However, the DM-associated oxidative stress, mitochondrial dysfunction and inflammation may enforce AF development and re-entry. The accumulation and infiltration of EAT and thrombus formation could also have an effect. Thus, DM patients with damaged cardiac conductivity and AF subjects with uncontrolled glycaemia should be diagnosed early and treated. In this sense, anti-diabetic treatments such as metformin, PPAR-α agonists, and mainly SGLT2 inhibitors, could be of great use. More studies analyzing the direct and indirect associations, as well as the potential mechanisms of interaction are needed to establish the causal relationship between AF and DM, and related comorbidities.

## Data Availability

No new data were created or analyzed in this study. Data sharing is not applicable to this article.

## Abbreviations

Atrial fibrillation (AF), type-2 diabetes mellitus (T2DM), non-vitamin-K oral anticoagulants (NOACs), peroxisome proliferator-activated receptor alpha (PPAR-α), adenosine triphosphate (ATP), mitochondrial uncoupling protein-3 (UCP-3), reactive oxygen species (ROS), advanced glycated end products (AGE), nuclear factor kappa β pathway (NF-κB), interleukin-6 (IL-6), tumor growth factor-β (TGF-β), tumor growth factor-α (TGF-α), tumor necrosis factor-alpha (TNF-α), calcium (Ca^2+^), sarcoplasmic reticulum (SR), ryanodine receptors (RyR2s), Ca^2+^/calmodulin kinase type II (CaMKII), calcineurin/nuclear factor of activated T cells (NFAT), L-type Ca^2+^ current (IcaL), heart failure with preserved ejection fraction (HFpEF), renin–angiotensin–aldosterone system (RAAS), epicardial adipose tissue (EAT), interleukin-1β (IL-1β), matrix metalloproteinases (MMP), tissue-plasminogen activator (t-PA), tissue plasminogen activator inhibitor (PAI-1), nitric oxide (NO), low-density lipoprotein (LDL), tissue factor (TF), complement (C3), glucagon-like peptide-1 receptor agonists (GLP-1 RA), Dipeptidyl peptidase-4 (DPP-4) inhibitors, sodium glucose co-transporter-2 inhibitor (iSGLT2).
